# Dynamic cellular heterogeneity revealed through a time-resolved single-cell atlas: assessment of porcine intestinal organoids as an in vitro model for deoxynivalenol and zearalenone

**DOI:** 10.1186/s40104-026-01424-9

**Published:** 2026-06-03

**Authors:** Tae Hong Kang, Jung Woong Yoon, Seung Joon Lim, Chae Hyun Lee, Yo Han Lee, Suhyeon Yun, Heejun Jung, Hyun Jun Jang, Tae Hyun Kim, Sang In Lee

**Affiliations:** 1 Department of Animal Science and Biotechnology, Kyungpook National University, Sangju-Si, Gyeongsangbuk-Do 37224 Republic of Korea; 2 Biopharmaceutical Materials Department, Korea Polytechnics, 15 Gukchaebosang-Ro 43-Gil, Seo-Gu, Daegu, Republic of Korea; 3 Center for Industrialization of Agricultural and Livestock Microorganisms, Jeongeup, 56212 Korea; 4 Department of Animal Science, The Pennsylvania State University, University Park, PA 16802 USA; 5 Research Institute for Innovative Animal Science, Kyungpook National University, Sangju-Si, Gyeongsangbuk-Do 37224 Republic of Korea

**Keywords:** Deoxynivalenol, Organoid, Single cell sequencing, Zearalenone

## Abstract

**Background:**

Intestinal epithelial cells are supported by dynamic cellular heterogeneity, which is critical for maintaining intestinal homeostasis. Recently, intestinal organoid models have gained attention as in vitro platforms because they can recapitulate the structural, functional, and cellular complexity of the small intestine. In this study, we developed porcine intestinal organoids and investigated time-dependent transcriptomic changes using single-cell RNA sequencing. Furthermore, to assess their applicability as an in vitro toxicity model, the organoids were exposed to the mycotoxins deoxynivalenol and zearalenone.

**Results:**

The established organoids exhibited stable long-term culture up to passage 10 (32 d) and showed high genetic similarity to native small intestinal tissue across three regions: the duodenum, jejunum, and ileum. In various intestinal epithelial cell types, including transit-amplifying cells, enteroendocrine cells, goblet cells, Paneth cells, and other epithelial cell types, were identified in the organoids. Single-cell RNA sequencing classified the organoids into multiple distinct cell populations, including stem cells, transit-amplifying cells, secretory progenitors, enterocytes, enteroendocrine cells, goblet cells, and Paneth cells, demonstrating dynamic cellular heterogeneity. The organoids also recapitulated key intestinal functions, such as nutrient absorption and epithelial barrier formation, similar to those of the native small intestinal epithelium. Under these conditions, the cytotoxic effects of deoxynivalenol and zearalenone were evaluated. Treatment with these mycotoxins resulted in decreased cell viability, impaired intestinal barrier function, and altered rates of proliferation and differentiation, including those of enteroendocrine, goblet, and Paneth cell populations.

**Conclusion:**

This study provides fundamental insights into the growth and differentiation of small intestinal epithelial cells by analyzing timeline-specific organoids using single-cell sequencing. Additionally, it evaluates the toxicity of mycotoxins under conditions that closely resemble those of the small intestine, providing more physiologically relevant data than existing in vitro models and serving as a reliable toxicity assessment model.

**Supplementary Information:**

The online version contains supplementary material available at 10.1186/s40104-026-01424-9.

## Background

Small intestinal epithelial cells exhibit considerable plasticity, which enables self-renewal, proliferation, differentiation, and dedifferentiation driven by intestinal stem cells (ISCs) along the crypt-to-villus axis [1]. In the crypt, ISCs are strictly regulated by niche signals that control their self-renewal and differentiation into absorptive and secretory cell types. The small intestinal epithelium is composed of a single layer of cells that are tightly connected through specialized intercellular junctions, including tight junctions, adherens junctions, and gap junctions. This highly organized epithelial structure maintains tissue integrity and regulates paracellular permeability. The small intestinal epithelium plays a crucial role in efficient nutrient uptake while simultaneously serving as a protective barrier against harmful antigens and pathogens [2, 3].

In the porcine industry, mycotoxins are major harmful factors that cause significant economic losses [4]. Mycotoxins are secondary metabolites produced by fungi and are widespread globally, particularly in feedstuff derived from grains, including corn, wheat, barley, and oats [5, 6]. Among the various mycotoxins, ZEA and DON, produced by *Fusarium* species, have the highest detection rates in animal feed [7]. These toxins first encounter the small intestinal epithelium and are rapidly absorbed there [8, 9]. Pigs are the most susceptible to DON. Upon ingestion, DON induces inflammation, disrupts the intestinal mucosal barrier, impairs nutrient absorption, and increases the risk of opportunistic infections in the small intestine [10]. ZEA is known to disrupt intestinal structure and function and to induce inflammation, oxidative stress, and hormonal imbalances [11].

Intestinal organoids, generated from ISCs, form three-dimensional (3D) cultures that reproduce the crypt–villus architecture of the small intestine. These systems exhibit essential biological properties, including self-renewal, self-organization, and multilineage differentiation into epithelial cell populations such as stem cells, transit-amplifying (TA) cells, enterocytes, enteroendocrine cells, goblet cells, and Paneth cells [12]. Given their similarity to the native small intestine in terms of cellular heterogeneity, plasticity, and structural organization, intestinal organoids have attracted increasing attention in farm animal studies, including in bovine, porcine, and chicken models. They have been widely applied as in vitro systems for investigating toxicity, viral infections, and disease pathogenesis [13–15]. However, despite their remarkable plasticity, most existing studies have been limited to characterization, and further investigations are needed to examine their effects on diverse cell types in porcine intestinal organoids.

Recent single-cell RNA sequencing (scRNA-seq) has emerged as a powerful tool for investigating cellular heterogeneity, reconstructing transcriptional trajectories, and exploring cancer individuality [16]. The small intestinal epithelium contains diverse cell types, and bulk RNA sequencing does not adequately capture their cellular heterogeneity. In contrast, scRNA-seq enables transcriptomic profiling at the individual cell level and is therefore well suited for accurately characterizing the heterogeneity of intestinal epithelial cells [17]. However, scRNA-seq has not been applied in small intestinal organoid research in livestock. Most studies have focused on cell type characterization, evaluating the suitability of two-dimensional (2D) culture systems for intestinal functions, and examining the impact of viral infection models [18].

To address this gap in the literature, this study aimed to evaluate whether the developed porcine intestinal organoids recapitulate the temporal development of the native small intestine and to characterize their dynamic cellular heterogeneity. We further confirmed that these organoids exhibit key features of the small intestinal epithelium, including nutrient uptake functions (glucose, amino acids, and fatty acids) and intestinal barrier integrity. Furthermore, we assessed the effects of DON and ZEA on cytotoxicity, proliferation rates, and differentiation into enteroendocrine cells, goblet cells, and Paneth cells. Our findings demonstrate that the growth and differentiation dynamics of these organoids are comparable to those of native small intestinal epithelial cells, reflecting essential aspects of in vivo intestinal physiology. Given their high biological relevance, these organoids represent a powerful in vitro model for studies of toxicity, cellular differentiation, and intestinal disease.

## Methods

### Preparation of L-WRN organoid medium

L-WRN (ATCC^®^) cells, selected using a G418 sulfate (Biosesang, Yongin-si, Republic of Korea), were seeded into culture plates and incubated in DMEM/F12 (Thermo Fisher Scientific, Wilmington, DE, USA) containing 1% penicillin–streptomycin (Thermo Fisher Scientific, Wilmington, DE, USA) and 20% FBS. Cells were cultured until they reached 80%–90% confluence and then cultured for 3 d. The medium was collected daily and filtered using a syringe filter (0.2 μm). The L-WRN culture medium was mixed at a 1:1 ratio with fresh DMEM/F12 containing the same supplements, and further supplemented with 500 nmol/L A83-01 (Tocris Bioscience), 50 ng/mL hEGF (Thermo Fisher Scientific, Wilmington, DE, USA), 10 μmol/L Y-27632 (MedChemExpress), 1 × B-27 supplement (Thermo Fisher Scientific, Wilmington, DE, USA), and 1 × N-2 supplement (Thermo Fisher Scientific, Wilmington, DE, USA).

### Porcine intestinal organoid culture and mycotoxin treatment

Small intestinal crypts were obtained from three-way crossbred gilts (Landrace × Yorkshire × Duroc). Briefly, small intestinal tissue was washed with PBS containing 1% PS, sliced, and incubated with dissociation solution (StemCell Technologies, Vancouver, BC, Canada) at room temperature (RT) for 1 h. After centrifugation at 244 × *g* for 5 min, the supernatant was discarded, and the tissue pellet was washed with DMEM/F12. The cell pellet was resuspended in DMEM/F12 and filtered sequentially through 100 μm and 70 μm cell strainers. After centrifugation at 244 × *g* for 5 min, the crypt pellet was resuspended in L-WRN organoid medium containing matrigel (Corning, USA) at a 1:1 ratio. The medium was replaced every 3 d. Following this, organoids were resuspended in L-WRN medium and DMSO at a 1:9 ratio and then incubated at 4 °C for 10 min, −20 °C for 1 h, and −75 °C overnight before storage in liquid nitrogen. DON (Sigma-Aldrich, Burlington, MA, USA) was applied at concentrations of 0.25, 0.5, 1, 2, 4, and 8 μmol/L for 24 h; and ZEA (Sigma-Aldrich, Burlington, MA, USA) was applied at 2.5, 5, 10, 20, 40, and 80 μg/mL for 48 h to porcine intestinal organoid cells.

### Analysis of scRNA-seq

Jejunum derived organoids cultured for 1, 3, and 5 d were treated with TrypLE (Thermo Fisher Scientific, Wilmington, DE, USA) containing 10 μmol/L Y-27632 for 20 min, with pipetting every 5 min to obtain single-cell suspensions. Dead cells were removed using a dead cell removal kit (Miltenyi Biotec, Bergisch Gladbach, Germany). Single cells were washed with PBS containing 0.04% BSA and counted using a Countess II cell counter (Thermo Fisher, Waltham, MA, USA). To obtain single-cell RNA-sequencing libraries, Chromium Next GEM Single Cell 3′ reagent kit v3.1 (10X Genomics, Pleasanton, CA, USA) was used according to the manufacturer’s instructions. Cells were diluted into the Chromium Next GEM Chip G to yield a recovery of approximately 5,000 cells per sample. Libraries were sequenced on the NovaSeq 6000 sequencer (Illumina, San Diego, CA, USA), yielding an average of at least 60,000 reads per single cell. The raw read data can be accessed from the Gene Expression Omnibus (GEO) under accession number GSE315817.

### Single-cell RNA sequencing data analysis

Sequencing reads were processed using Cell Ranger (v9.0.0) (10X Genomics) with the porcine reference transcriptome Sscrofa11.1 obtained from Ensembl. From the gene expression matrix, downstream analyses were performed in R (v4.2.2). The filtered gene expression matrices generated by Cell Ranger were used for quality control, data filtering, clustering, visualization, and differential expression analysis using the Seurat package (v5.3.0) [19]. Genes detected in fewer than 500 cells and cells containing fewer than 200 unique molecular identifiers (UMIs) were excluded. Cells exhibiting mitochondrial gene-derived UMIs exceeding 5% of total UMIs were also removed. Data were normalized using the LogNormalize method, followed by scaling of gene expression values while regressing out total UMI counts and the proportion of mitochondrial gene expression. Principal component analysis (PCA) was performed on the normalized expression matrix, and the first 18 principal components were used for downstream clustering and visualization. Clustering was performed at a resolution of 0.8, and results were visualized using uniform manifold approximation and projection (UMAP). Gene expression patterns were assessed using FeaturePlot, ViolinPlot, DotPlot, and VolcanoPlot functions.

### Cell viability

Organoids were dissociated using TrypLE supplemented with 10 μmol/L Y-27632 at 37 °C in a CO_2_ incubator for 20 min, with pipetting every 5 min. Cells were centrifuged at 244 × *g* for 5 min, and resuspended in L-WRN organoid medium mixed with Matrigel at a 1:1 ratio. Seventy cells in a 10 μL volume were seeded into each well of a 96-well plate. Cell viability was assessed using a WST-8 assay (Abcam, Cambridge, UK), and absorbance was measured at 450 nm after 2 h using a microplate reader.

### Paraffin embedding of porcine intestinal organoids and H&E staining

For paraffin embedding, organoids were fixed in 4% paraformaldehyde (PFA) for 30 min and dehydrated sequentially in 70%, 80%, 90%, and 100% ethanol for 30 min each. For clearing, organoids were incubated in xylene for 1 h and subsequently infiltrated with pre-warmed paraffin wax at 60 °C for 1 h prior to embedding. Organoid sections were incubated on a 50 °C hot plate before staining. For staining, a Hematoxylin and Eosin (H&E) staining kit was used according to the manufacturer’s instructions. Briefly, sections were deparaffinized, rehydrated in distilled water, and stained with hematoxylin for 5 min. Sections were then washed in distilled water, treated with bluing reagent, and rinsed again in distilled water. Subsequently, sections were counterstained with eosin for 2 min, dehydrated in 100% ethanol, and mounted.

### Immunostaining of porcine intestinal organoids

Organoids were fixed in 4% PFA and washed three times with PBS for 5 min each. Organoids were permeabilized with 0.5% Triton X-100 in PBS, followed by blocking in 5% normal goat serum and 0.3% Triton X-100 in PBS. Organoids were incubated overnight at 4 °C with appropriate primary antibodies, such as F-actin (Bioss, Massachusetts, USA), Chromogranin A (Abcam, Cambridge, UK), Lysozyme (Abcam, Cambridge, UK), Mucin-2 (MyBioSource, San Diego, CA, USA), and Zonula occludens-1 (Thermo Fisher Scientific, Wilmington, DE, USA). Organoids were then incubated with the appropriate secondary antibodies at RT for 1 h in the dark and mounted using an antifade mounting medium containing DAPI. Imaging was performed using a confocal microscope (LSM 900 with Airyscan 2; Zeiss, Oberkochen, Germany).

### 5-Ethynyl-2′-deoxyuridine (EdU) staining

Cell proliferation was assessed using a cell proliferation 5-ethynyl-2′-deoxyuridine (EdU) imaging kit (MyBioSource, San Diego, CA, USA). Staining was performed in accordance with the manufacturer’s protocol. Prior to harvesting, organoids were incubated with 10 μmol/L EdU for 2 h. Following incubation, the culture medium was removed and the cells were fixed with 4% paraformaldehyde for 15 min. Organoids were washed three times with 1 × BSA working solution for 5 min each and subsequently permeabilized for 15 min. Cells were washed twice with 1 × BSA working solution for 5 min and incubated with the Click-iT reaction mixture for 30 min in the dark. Finally, organoids were washed with BSA wash solution and mounted using an antifade mounting medium containing DAPI.

### Quantitative real-time PCR

Total RNA was extracted from organoid cells using the AccuPreP universal RNA extraction kit (Bioneer, Daejeon, Republic of Korea). cDNA was synthesized using the DiaStar™ RT kit (SolGent, Daejeon, Republic of Korea). Primers were designed using Primer3 (primer3.ut.ee). The following steps were used for qPCR: 95 °C for 15 s, followed by 56–63.5 °C for 15 s, and 72 °C for 15 s. Gene expression levels were normalized using glyceraldehyde-3-phosphate dehydrogenase (*GAPDH*) as the housekeeping gene.

### Apical-out organoid formation

Organoid cells were cultured for 3 d. After discarding the culture medium, organoid cells were incubated with 5 mol/L EDTA in PBS for 1 h at 4 °C. Cells were centrifuged at 244 × *g* for 5 min and washed with DMEM/F12. After centrifugation, the cells were incubated in L-WRN organoid medium without Matrigel. Apical-out formations were clearly observed for up to 2 d post-seeding.

### Permeability assay

Organoids were washed with non-phenol red DMEM (Thermo Fisher Scientific, Wilmington, DE, USA), harvested, and incubated in nonphenol red DMEM including concentration at 25 ng/mL of fluorescein isothiocyanate (FITC)-dextran (4 kDa) for 30 min in the dark at RT. Imaging was performed using a fluorescence microscope (Korealabtech, Gyeonggi-Do, Republic of Korea).

### Amino acid uptake assay

After removing the culture medium, phenol red-free DMEM was added, and Matrigel was dissociated by pipetting. Organoids were centrifuged at 244 × *g* for 5 min. The resulting pellet was transferred to 15 mL conical tubes and washed twice with prewarmed Hanks’ balanced salt solution (HBSS; Sigma-Aldrich, Burlington, MA, USA). The organoids were subsequently incubated for 5 min at 37 °C in a CO_2_ incubator. Following removal of the supernatant, organoids were exposed to a prewarmed boronophenylalanine (BPA) uptake solution composed of HBSS, BPA solution, and BPA dilution buffer, and incubated again for 5 min at 37 °C in a CO_2_ incubator. The supernatant was discarded, and organoids were washed three times with prewarmed HBSS. Following the final wash, organoids were treated with a prewarmed working solution consisting of HBSS and probe solution (Dojindo, Kumamoto-ken, Japan) and incubated for an additional 5 min at 37 °C in a CO_2_ incubator. The organoid cells were then stained with NucSpot^®^ 470 (Biotium, Fremont, CA, USA) and representative images were acquired using a confocal microscope (LSM 900 with Airyscan 2).

### Glucose uptake assay

After the culture medium was removed, phenol red-free DMEM was added, and the Matrigel was mechanically disrupted by pipetting. Organoids were subsequently centrifuged at 244 × *g* for 5 min, washed with glucose- and serum-free medium, and incubated for 15 min at 37 °C in a CO_2_ incubator. Following removal of the supernatant, organoids were treated with a prewarmed probe solution consisting of serum-free medium mixed with a glucose uptake probe (Dojindo, Kumamoto-ken, Japan) and incubated for an additional 15 min at 37 °C in a CO_2_ incubator. The supernatant was discarded, and organoids were washed with WI solution and incubated at RT for 5 min. Organoids were then stained with DAPI. Representative images were acquired using a confocal microscope.

### Fatty acid uptake assay

After removal of the culture medium, organoids were washed with non-phenol red DMEM and incubated with 5 μmol/L BSA in C1-BODIPY-C12 (Invitrogen, Wilmington, DE, USA) at 37 °C for 30 min. Organoids were stained with DAPI (Vector Laboratories, Burlingame, CA, USA) and mounted on glass slides. Images were obtained using a confocal microscope.

### Quantification of CHGA, MUC2, and LYZ in porcine intestinal organoids

Organoids were dissociated using TrypLE containing 10 μmol/L Y-27632 at 37 °C in a CO_2_ incubator for 20 min, with pipetting every 5 min. Cells were fixed for 15 min and permeabilized for 30 min. Cells were blocked for 1 h at RT and then incubated overnight with appropriate antibodies. Secondary antibodies were then added, and the mixture was incubated for 1 h in the dark, followed by three washes. Positive cells were detected using a BD FACSVerse flow cytometry system (BD Biosciences, San Jose, CA, USA).

### Annexin V/PI staining

Organoids were dissociated in TrypLE containing concentration at 10 μmol/L Y-27632 at 37 °C in a CO_2_ incubator for 20 min, with pipetting every 5 min. After centrifuging, cell pellet was washed from PBS, and incubated with V (Thermo Fisher Scientific, Wilmington, DE, USA) and 100 μg/mL of propidium iodide (PI) in 1 × annexin V buffer for 15 min in dark. To detect positive cells, the BD FACSVerse flow cytometry system (BD Biosciences, San Jose, CA, USA) was utilized.

### Statistical analysis

Statistical analyses were performed using SAS software (v9.4), with significance between experimental and control groups determined using a *t*-test. Results are expressed as means ± standard deviation from three independent experiments. Statistical significance was defined as: ^*^P < 0.05, ^**^P < 0.01, and ^***^P < 0.001. Cell viability data were tested for one-way ANOVA. Additional comparisons among groups were performed using Tukey’s multiple comparison test.

## Results

### Development and characterization of porcine intestinal organoids

**Fig. 1:**
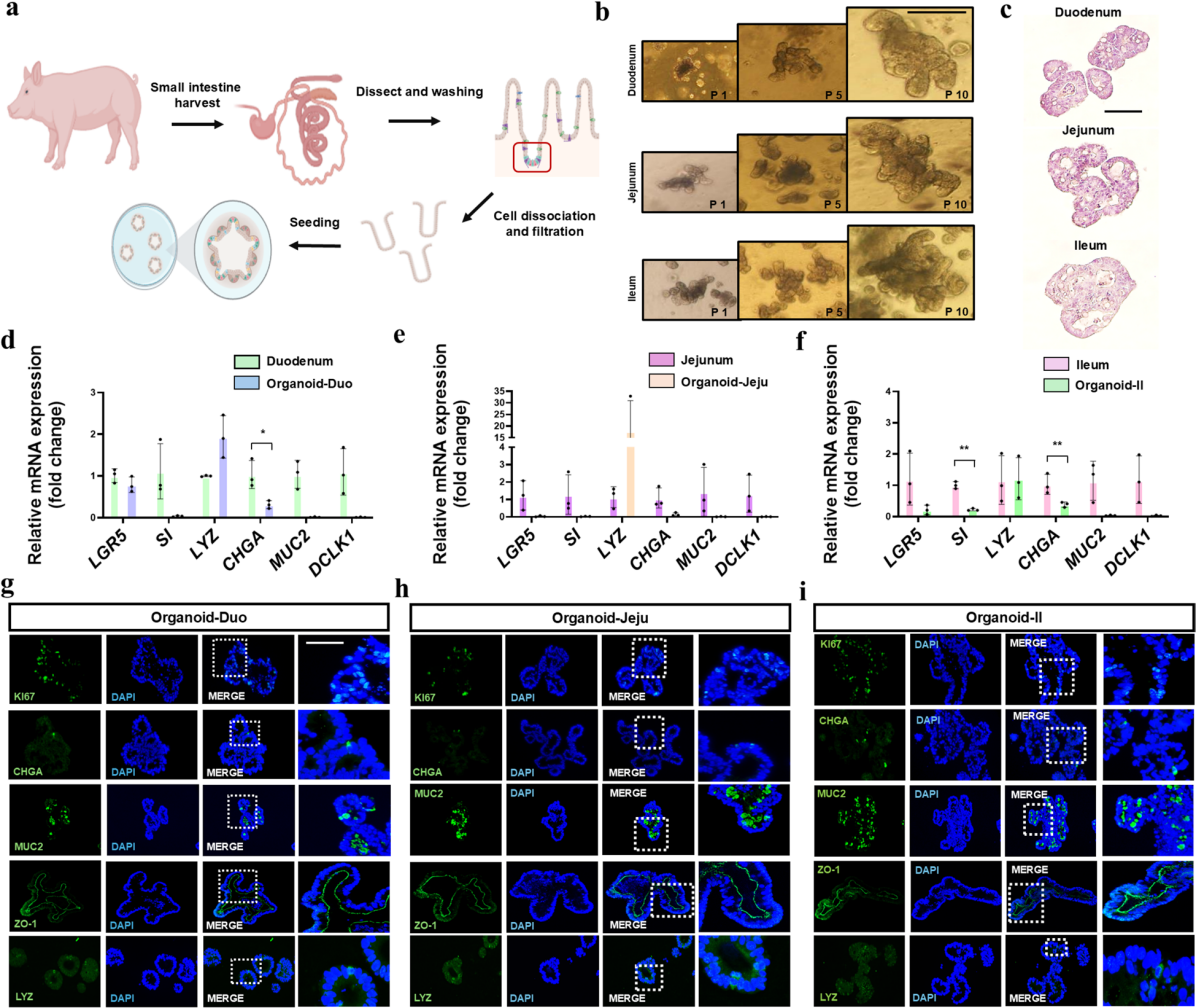
Porcine intestinal organoid culture and characterization. **a** Schematic illustration of the organoid culture of porcine intestinal organoids derived from the duodenum, jejunum, and ileum. **b** Development of porcine intestinal organoids from passage 1 to passage 10 in the duodenum, jejunum, and ileum. **c** Hematoxylin and eosin staining of duodenal, jejunal, and ileal organoids. **d** Relative mRNA expression of *LGR5*, *SI*, *LYZ*, *CHGA*, *MUC2*, and *DCLK1* in duodenal organoids compared with native duodenum. **e** Relative mRNA expression of *LGR5*, *SI*, *LYZ*, *CHGA*, *MUC2*, and *DCLK1* in jejunal organoids compared with the native jejunum. **f** Relative mRNA expression of *LGR5*, *SI*, *LYZ*, *CHGA*, *MUC2*, and *DCLK1* in ileal organoids compared with the native ileum. **g** Immunostaining of KI67, CHGA, MUC2, LYZ and ZO-1 duodenal organoids. **h** Immunostaining of KI67, CHGA, MUC2, LYZ and ZO-1 in jejunal organoids. **i** Immunostaining of KI67, CHGA, MUC2, LYZ and ZO-1 in ileal organoids. Organoid images were obtained using an optical microscope. Immunostaining images were obtained using a confocal microscope. Nuclei were stained with 4′,6-diamidino-2-phenylindole (DAPI; blue). Scale bars = 20 and 50 μm. Error bars indicate the standard deviation (SD) of analyses performed in triplicate. ^*^P < 0.05; ^**^P < 0.01; ^***^P < 0.001

The porcine intestinal organoid culture procedure, from intestinal stem cell isolation to organoid culture, is presented in Fig. 1a. Dissociated intestinal stem cells derived from the duodenum (Duo), jejunum (Jeju), and ileum (Il) were cultured to produce organoids that were stably maintained for an extended period, from passage 1 (P1, 5 d) to passage 10 (P10, 32 d). These organoids exhibited crypt–villus–like structures (Fig. 1b and c). To confirm the cellular heterogeneity of the developed organoids, mRNA expression levels of intestinal epithelial cell type–specific markers were analyzed and compared with those of the corresponding regions of the native small intestine (Duo, Jeju, and Il). The analyzed markers included the intestinal stem cell marker leucine rich repeat containing G protein-coupled receptor 5 (*LGR5*), the enterocyte marker sucrase-isomaltase (*SI*), the enteroendocrine cell marker chromogranin A (*CHGA*), the goblet cell marker mucin-2 (*MUC2*), and the tuft cell marker doublecortin-like kinase 1 (*DCLK1*). Between Duo and the corresponding organoids, *CHGA* expression was significantly decreased in organoids, whereas the expression of other cell markers, such as *LGR5*, *SI*, *LYZ*, *MUC2*, and *DCLK1*, was not significantly different (Fig. 1d). No significant differences in cell-type marker expression were observed between Jeju and the corresponding organoids (Fig. 1e). However, in the Il organoids, the relative expression levels of *SI* and *CHGA* were significantly decreased (Fig. 1f). To characterize organoids derived from Duo, Jeju, and Il, the expression of the transit-amplifying cell and proliferating cell marker KI67, CHGA, MUC2, LYZ and the epithelial cell marker zonula occludens-1 (ZO-1) was examined. Expression was observed in all organoids (Fig. 1g–i). These results indicate that the developed organoids can be stably maintained for longer periods and exhibit heterogeneity similar to intestinal epithelial cell types.

### Clustering of porcine intestinal organoids

**Fig. 2:**
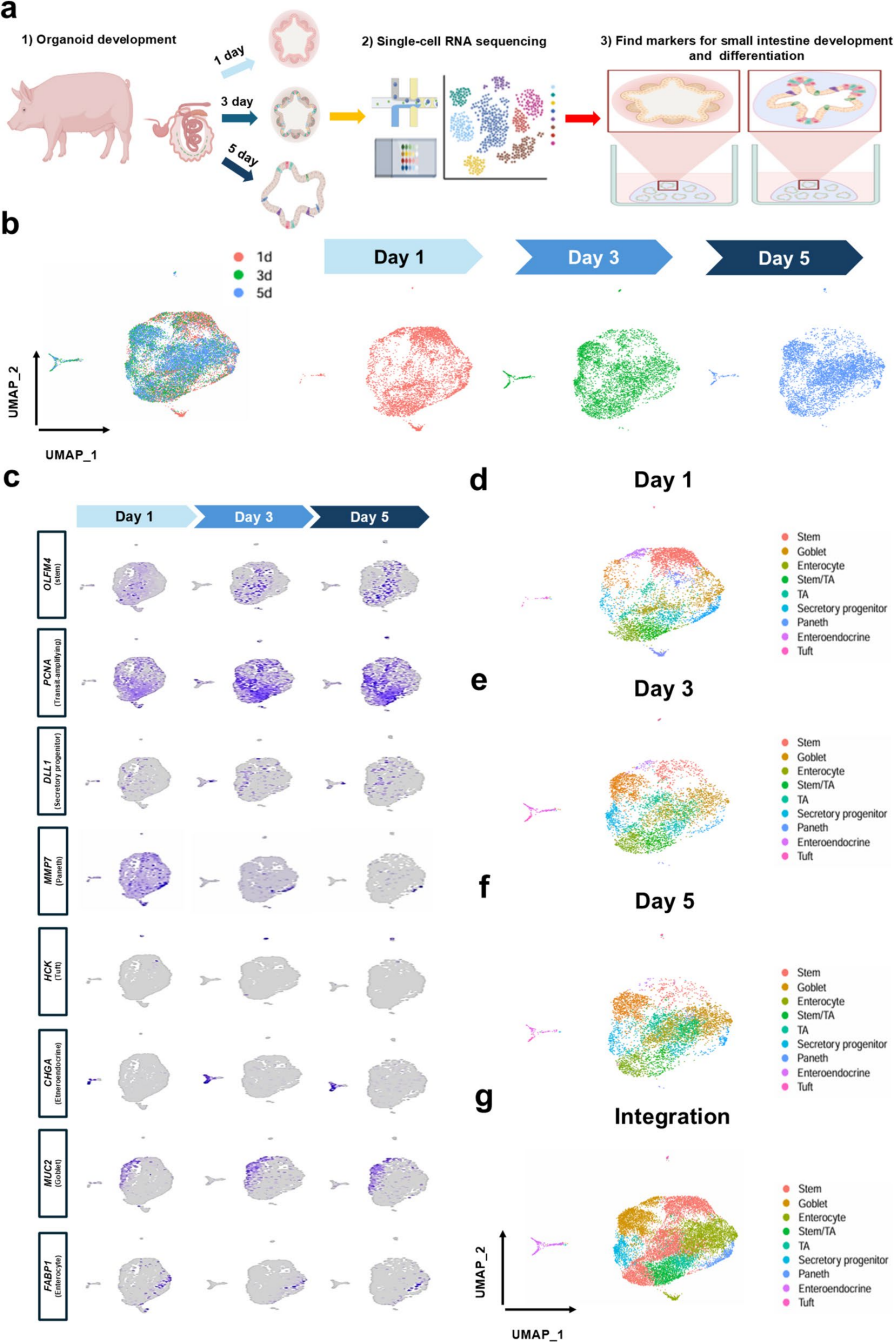
Identification of intestinal epithelial cell types in porcine intestinal organoids. **a** Schematic illustration of the scRNA-seq analysis procedure for jejunum derived organoids. **b** Uniform manifold approximation and projection (UMAP) of porcine intestinal organoids at different time points. **c** Feature plots of *OLFM4*, *PCNA*, *DLL1*, *MMP7*, *HCK*, *CHGA*, *MUC2*, and *FABP*1 in porcine intestinal organoids at different time points. **d** UMAP plot of intestinal organoid cells after 1 day of culture. **e** UMAP plot of intestinal organoid cells after 3 days of culture. **f** UMAP plot of intestinal organoid cells after 5 days of culture. **g** UMAP plot of integrated organoid cells from all time points, broadly clustered into nine cell types: stem (red), goblet (orange), enterocyte (yellow-green), stem/TA (green), TA (cyan), secretory progenitor (light blue), Paneth (blue), enteroendocrine (purple), and tuft (pink)

To determine whether the developed organoids recapitulate small intestinal development and differentiation patterns, scRNA-seq was performed on organoids cultured for 1, 3, and 5 d. The scRNA-seq procedure is shown in Fig. 2a. After filtering out dead and low-quality cells based on quality control thresholds (gene count < 200 or mitochondrial RNA > 10%), the final dataset comprised 15,317 cells across the three culture time points. UMAP visualization revealed changes in cell population distributions across the three time points (Fig. 2b). Furthermore, FeaturePlot analysis was used to characterize intestinal organoid cell types. Stem cells were characterized by the expressions of *OLFM4*, *LGR5*, and *ASCL2*; TA cells by *PCNA*, secretory progenitor cells by *DLL1*; enterocytes by *FABP2*, *ALDOB*, and *APOA1*; goblet cells by expression of *MUC2*, *AGR2* and *SPDEF*; enteroendocrine cells by *CHGA*; tuft cells by *HCK*; and Paneth cells by *MMP7* (Fig. 2c). Based on distinct cell-type–specific marker expression patterns, the clusters were annotated (Fig. 2d–g).

### Single-cell transcriptomics uncovers dynamic maturation and lineage progression of intestinal epithelial cells

**Fig. 3:**
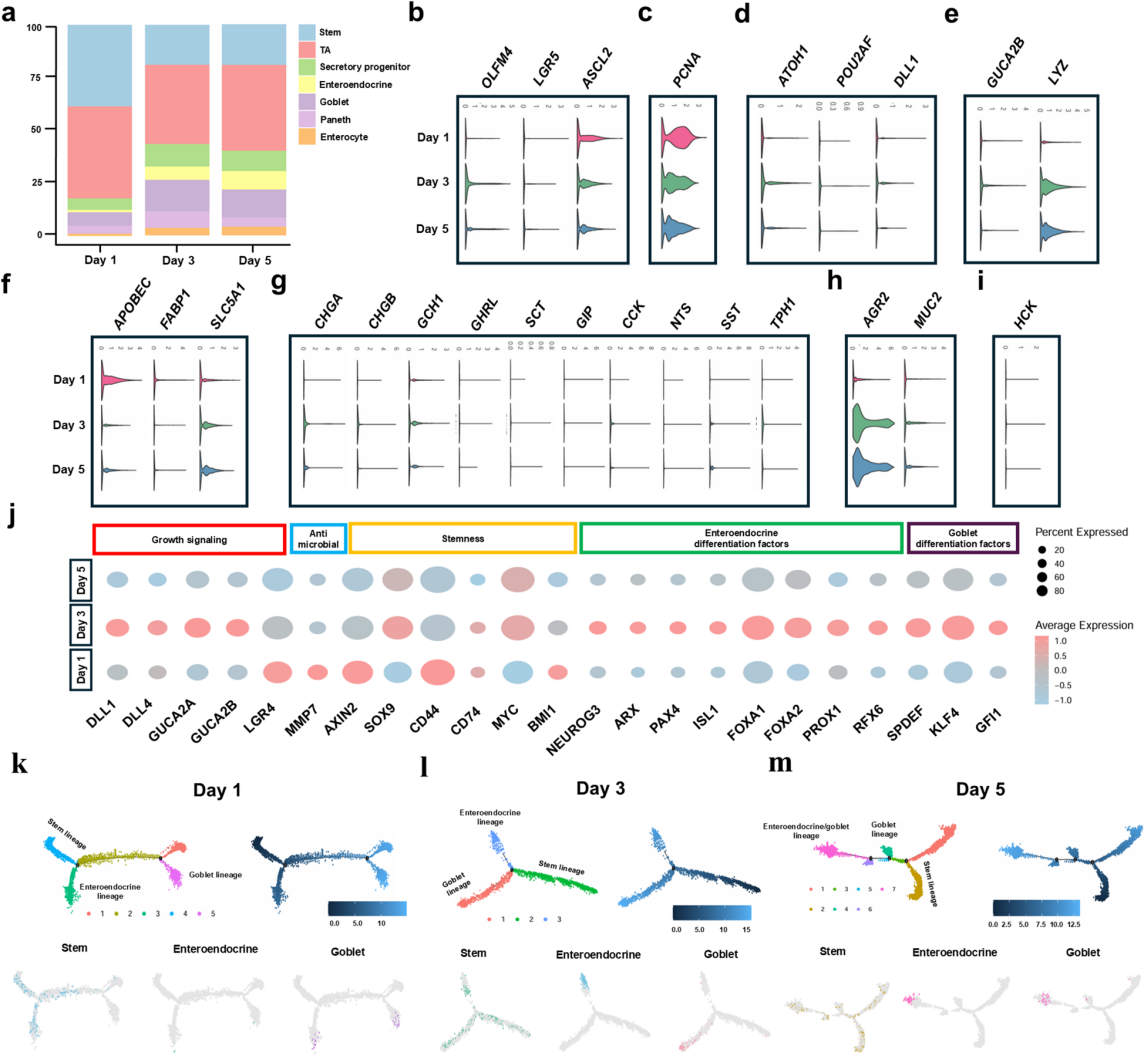
Heterogeneity in porcine intestinal organoid cells at different time points. **a** Changes in the composition of intestinal epithelial cell types (% of cells) at different time points. **b** Violin plot of stem cell marker genes at different time points. **c** Violin plot of TA marker genes at different time points. **d** Violin plot of secretory progenitor marker genes at different time points. **e** Violin plot of Paneth cell marker genes at different time points. **f** Violin plot of enterocyte marker genes at different time points. **g** Violin plot of enteroendocrine and subtypes marker genes at different time point. **h** Violin plot of goblet cell marker genes at different time point. **i** Violin plot of tuft cell marker genes at different time points. **j** Dot plot of stemness, antimicrobial, enteroendocrine differentiation factors, and goblet cell differentiation factors. **k**–**m** Differentiation trajectory analysis using monocle2 with ISCs and ISCs-derived cells colored according to differentiation trajectories at different time points

To characterize the maturation of various epithelial cell types, expression patterns of cell type–specific markers were examined using epithelial cell-type proportions, ViolinPlots, DotPlots, and trajectory analysis. The proportion of differentiated epithelial cell types increased over time (Fig. 3a). Stem cell markers (*OLFM4*, *LGR5*, and *ASCL2*), the transit-amplifying (TA) cell marker *PCNA*, secretory progenitor markers (*ATOH1*, *POU2AF1*, and *DLL1*), Paneth cell markers (*GUCA2B* and *LYZ*), enterocyte markers (*APOBEC*, *FABP1*, and *SLC5A1*), enteroendocrine cell and subtype markers (*CHGA*, *CHGB*, *GCH1*, *GHRL*, *SCT*, *GIP*, *CCK*, *NTS*, *SST*, and *TPH1*), goblet cell markers (*AGR2* and *MUC2*), and the tuft cell marker *HCK* were detected in the 1, 3, and 5 d groups (Fig. 3b–i). Furthermore, in each group, genes related to growth signaling (*DLL1*, *DLL4*, *GUCA2A*, *GUCA2B*, and *LGR4*), antimicrobial activity and Paneth cell maturation (*MMP7*), stemness (*AXIN2*, *SOX9*, *CD44*, *CD74*, *MYC*, and *BMI1*), enteroendocrine differentiation (*NEUROG3*, *ARX*, *PAX4*, *ISL1*, *FOXA1*, *FOXA2*, *PROX1*, and *RFX6*), and goblet cell differentiation (*SPDEF*, *KLF4*, and *GFI1*) were confirmed. In the 3 d group, genes related to growth signaling, enteroendocrine differentiation, and goblet cell differentiation were generally upregulated compared with the other groups; however, *LGR4* expression was higher in the 1 d group. Stemness markers *SOX9*, *CD74*, and *MYC* were increased in the 3 d group, whereas *AXIN2*, *CD44*, and *BMI1* were more highly expressed in the 1 d group. *MMP7* showed the highest expression in the 1 d group (Fig. 3j). Next, to examine cell type–specific changes in motif enrichment during intestinal epithelial differentiation, intestinal epithelial lineage cells were subset into stem, enteroendocrine, and goblet cell populations, and pseudotime trajectory analysis was performed using Monocle. Among the three time points, pseudotime progression from the stem lineage toward enteroendocrine and goblet lineages was most pronounced in the 3 d group (Fig. 3k–m). To examine gene expression patterns and associated functional and pathway enrichment across differentiation time points from stem cells to mature secretory cell types, we analyzed differentially expressed genes (DEGs), Gene Ontology (GO); biological process (BP), cellular component (CC), and molecular function (MF), and Kyoto Encyclopedia of Genes and Genomes (KEGG) pathway enrichment (Additional file 1: Fig. S1). These data indicate that the established porcine intestinal organoid mimics the dynamic heterogeneity of the native small intestinal epithelium.

### Assessment of small intestinal function in porcine intestinal organoids

**Fig. 4:**
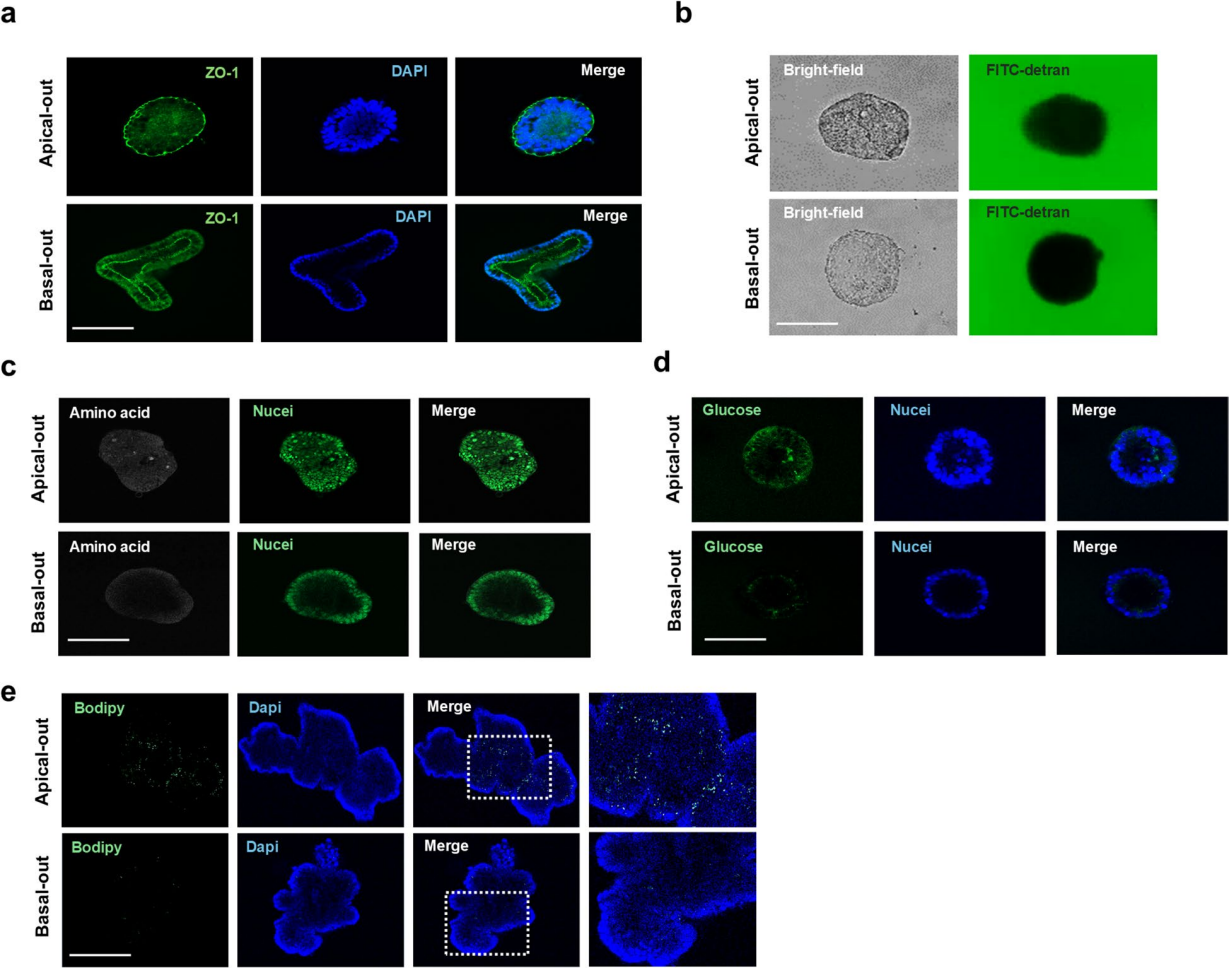
Assessment of intestinal function in porcine intestinal organoids. **a** Immunostaining of ZO-1 expression in basal-out and apical-out organoids. **b** Paracellular permeability analysis of porcine intestinal epithelial cells. **c** Amino acid uptake assay in porcine intestinal organoids. **d** Glucose uptake assay in porcine intestinal organoids. **e** Fatty acid uptake assay in porcine intestinal organoids. Nuclei were stained with DAPI (blue) and NucSpot (green). Scale bar = 50 μm

To validate whether the established intestinal organoids could mimic the native small intestinal epithelium, intestinal barrier function and nutrient absorption were examined. Conventional organoids typically exhibit an apical-in structure, with the apical membrane facing the lumen. To better recapitulate physiological conditions of the small intestine, apical-out organoids were generated and confirmed by ZO-1 expression (Fig. 4a). Barrier function in basal-out and apical-out organoids was assessed using FITC-dextran (4 kDa). In both models, FITC-dextran was not detected in the organoid lumen (Fig. 4b), indicating intact barrier function. To evaluate nutrient uptake, amino acid, glucose, and fatty acid analogs were applied to both basal- and apical-out organoids. Amino acids and glucose were absorbed in both models; however, apical-out organoids exhibited higher nutrient uptake efficiency compared with basal-out organoids. Similarly, fatty acid uptake was also higher in apical-out organoids (Fig. 4c–e). Additionally, organoids were assessed as an infection model. Aminopeptidase N (ANPEP) was expressed on the apical membrane of organoids (Additional file 2: Fig. S2). These results indicate that the established organoids function similarly to the native small intestinal epithelium, and that the apical-out form exhibits enhanced similarity. This model could also be utilized as an infection model.

### Cytotoxicity assessment of DON and ZEA using porcine intestinal organoids

**Fig. 5:**
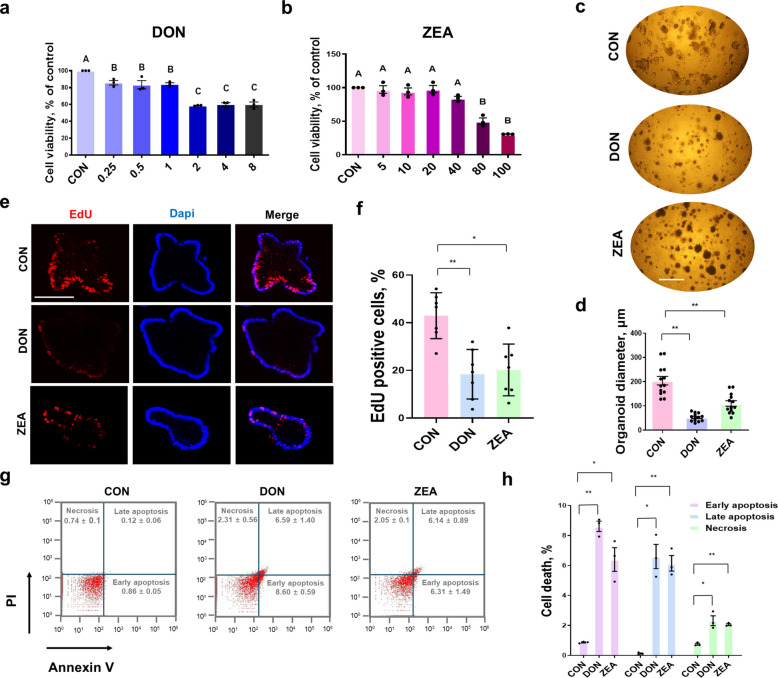
Assessment of cytotoxicity induced by DON and ZEA. **a** Cell viability after DON treatment in porcine intestinal organoids. **b** Cell viability after ZEA treatment in porcine intestinal organoids. **c** and **d** Morphological alteration induced by DON and ZEA. **e** and **f** Assessment of proliferating cell populations after DON and ZEA treatment (EdU; red, DAPI; blue). **g** and **h** Annexin V/PI staining to determine the percentage of apoptotic and necrotic cells after DON and ZEA treatment. Nuclei were stained with DAPI. Values are presented as mean ± SD. Experiments were conducted in triplicate. ^*^P < 0.05; ^**^P < 0.01; ^***^P < 0.001. Scale bar = 50 μm. Lowercase letters indicate significant differences between treatments, as determined by Tukey’s multiple range test

To assess cytotoxicity in porcine intestinal organoids, cell viability was examined. DON was tested at concentrations of 0.25, 0.5, 1, 2, 4, and 8 μmol/L, and ZEA was tested at 5, 10, 20, 40, 80, and 100 μg/mL. To evaluate the in vitro organoid model for DON and ZEA toxicity, concentrations were determined according to the inhibitor concentration 50 (Fig. 5a, b). In subsequent experiments, DON was tested at a concentration of 2 μmol/L, and ZEA was tested at 80 μg/mL. Following treatment with DON and ZEA, the crypt–villus-like structures were disrupted, and the organoids exhibited a significant decrease in diameter (Fig. 5c, d). To further evaluate the effects of DON and ZEA on cell viability, morphological alterations, and reduced diameter, the rates of cell proliferation and cell death were assessed. The proportion of proliferating cells was significantly lower in organoids treated with DON and ZEA than in the control group (Fig. 5e, f). In early apoptosis, the control group showed a ratio of 0.86% ± 0.05%, whereas the DON- and ZEA-treated groups exhibited ratios of 8.60% ± 0.59% and 6.31% ± 1.49%, respectively. The DON- and ZEA-treated groups showed a significant increase in early apoptosis compared to the control. Similarly, in late apoptosis and necrosis, the DON-exposed group showed ratios of 6.59% ± 1.40%, 2.31% ± 0.56%, respectively and the ZEA-treated group exhibited ratios of 6.14% ± 0.89% in late apoptosis and 2.05% ± 0.1% in necrosis, respectively. DON and ZEA treatment significantly increased all cell death populations compared to the control (Fig. 5g–h). These data indicate that DON and ZEA reduce stemness and increase cell death, leading to disruption of intestinal structure and dysfunction.

### Assessment of intestinal barrier function following DON and ZEA treatment

**Fig. 6:**
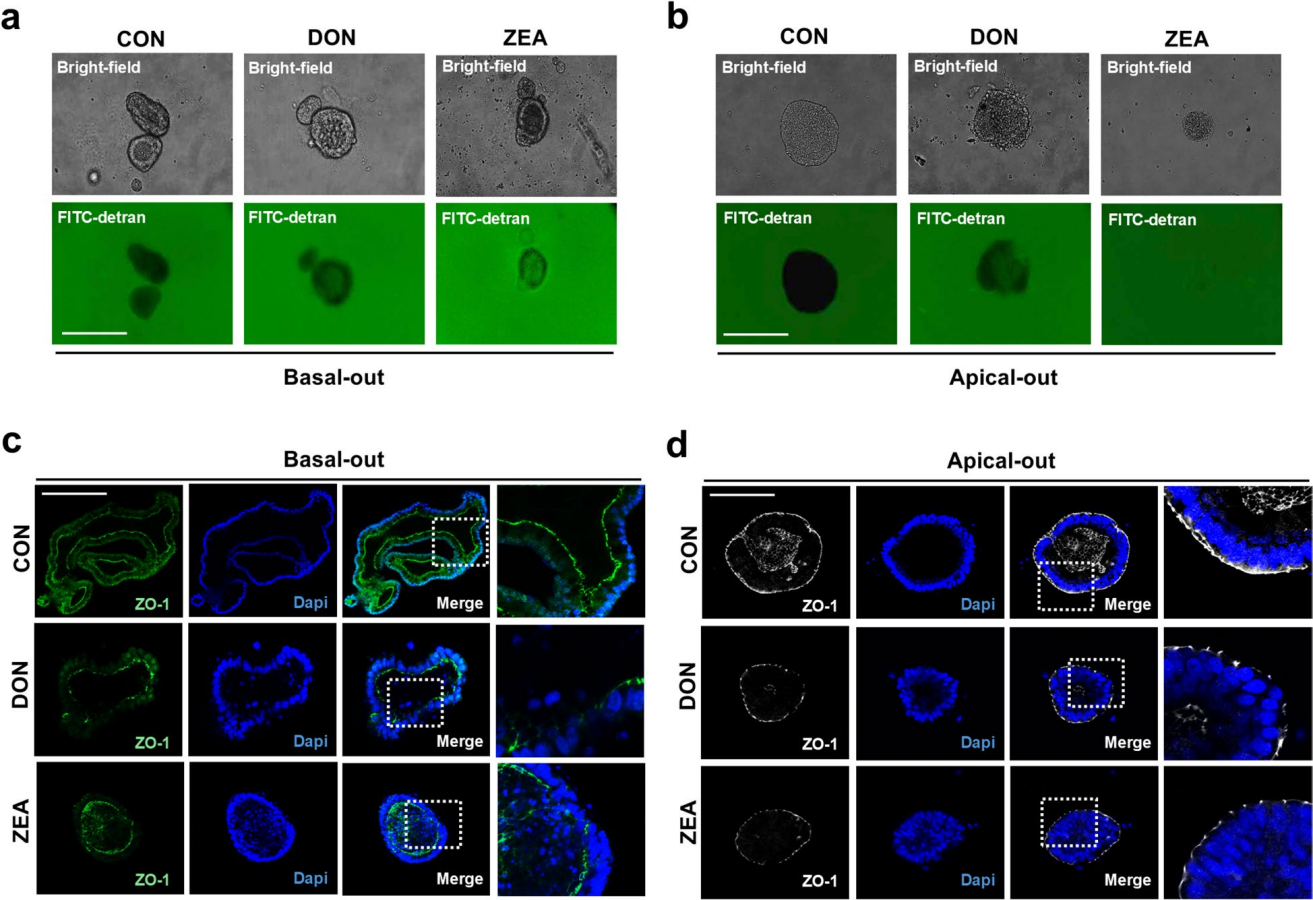
Assessment of intestinal barrier function following DON and ZEA treatment. **a** Paracellular permeability in basal-out organoids after DON and ZEA treatment. **b** Paracellular permeability in apical-out organoids after DON and ZEA treatment. **c** Immunostaining of ZO-1 expression after DON and ZEA treatment in basal-out organoid. **d** Immunostaining of ZO-1 expression in apical-out organoids after DON and ZEA treatment. Nuclei were stained with DAPI. Values are presented as mean ± SD. Experiments were conducted in triplicate. Scale bar = 50 μm

To examine intestinal barrier function following DON and ZEA treatment, a permeability assay using FITC-dextran (4 kDa) was performed in basal-out and apical-out configurations. In the basal-out form, the DON- and ZEA-treated groups showed increased FITC-dextran permeability into the lumen compared with the control group (Fig. 6a). Similarly, FITC-dextran permeability into the lumen increased in the apical-out form (Fig. 6b). To visually confirm ZO-1 expression in basal-out and apical-out forms, immunostaining was performed. In the control group, ZO-1 expression was continuously observed along the apical membrane, indicating that the epithelial junctions remained intact. However, the DON- and ZEA-treated group showed discontinuous ZO-1 expression along the apical membrane in the basal-out form (Fig. 6c). Similarly, in the apical-out form, the control group showed continuous ZO-1 expression in the apical membrane, whereas the DON- and ZEA-treated groups showed discontinuous ZO-1 expression (Fig. 6d). These results indicate that the developed organoid model is suitable for assessing intestinal barrier function in response to DON and ZEA.

### Effects of DON and ZEA on intestinal epithelial cell differentiation

**Fig. 7:**
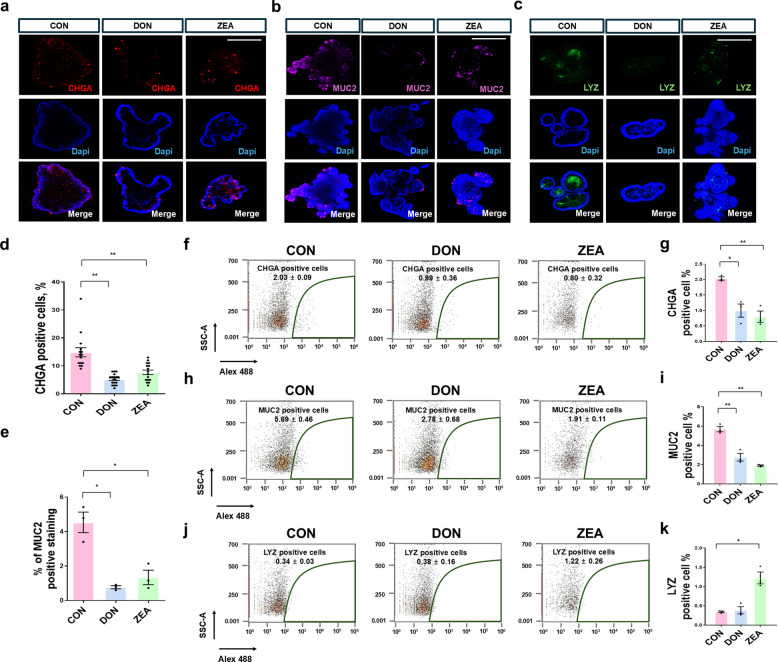
Effects of DON and ZEA on epithelial cell differentiation. **a** Immunostaining of CHGA-positive cells in porcine intestinal organoids after DON and ZEA treatment. **b** MUC2 immunostaining in porcine intestinal organoids after DON and ZEA treatment. **c** LYZ immunostaining in porcine intestinal organoids after DON and ZEA treatment. **d** Quantification of CHGA-positive cells in porcine intestinal organoids after DON and ZEA treatment. **e** Percentage of MUC2-positive cells in porcine intestinal organoids after DON and ZEA treatment. **f** and **g** Representative flow cytometry plots of CHGA-positive cells after DON and ZEA treatment. **h** and **i** Representative flow cytometry plots of MUC2-positive cells after DON and ZEA treatment. **j** and **k** Representative flow cytometry plots of LYZ positive cells after DON and ZEA treatment. Nuclei were stained with DAPI. Values are presented as mean ± SD. Experiments were conducted in triplicate. ^*^P < 0.05; ^**^P < 0.01; ^***^P < 0.001. Scale bar = 50 μm

To investigate the effects of DON and ZEA on intestinal epithelial differentiation, immunostaining was performed for CHGA, MUC2, and LYZ. The number of CHGA-positive cells was significantly reduced in the DON-treated group compared with the control, and a similar decrease was observed in the ZEA-treated group (Fig. 7a and d). MUC2-positive cells were also markedly decreased in both DON- and ZEA-treated groups relative to the control (Fig. 7b and e). LYZ-positive cells were present at very low levels in all organoids (Fig. 7c). Flow cytometry was used to quantify the proportions of CHGA-, MUC2-, and LYZ-positive cells in each group. The proportion of CHGA-positive cells was 2.03% ± 0.09% in the control group, 0.99% ± 0.36% in the DON-treated group, and 0.80% ± 0.32% in the ZEA-treated group, indicating reduced differentiation into enteroendocrine cells following DON and ZEA treatment (Fig. 7f and g). Similarly, the proportions of MUC2-positive cells were 5.69% ± 0.46%, 2.78% ± 0.68%, and 1.91% ± 0.11% in the control, DON-, and ZEA-treated groups, respectively, showing a significant reduction in goblet cell differentiation (Fig. 7h and i). In contrast, the proportion of LYZ-positive cells did not differ between the control and DON-treated groups, whereas ZEA treatment resulted in an increased proportion of LYZ-positive cells (Fig. 7j and k).

## Discussion

The small intestinal epithelium is composed of a diverse array of cell types, including ISCs, TA cells, progenitor cells, enterocytes, enteroendocrine cells, goblet cells, Paneth cells, and tuft cells. The intricate interplay among these cell types is essential for maintaining intestinal homeostasis, as each population contributes uniquely to processes such as proliferation along the crypt-to-villus axis, nutrient absorption, barrier function, hormone secretion, and immune regulation [20, 21]. Recently, 3D organoids derived from ISCs have been developed; these organoids mimic the crypt–villus structure and comprise diverse epithelial cell types in livestock [22, 23]. Maintenance of their structural features and cellular heterogeneity requires various growth factors and small molecules, including noggin, R-spondin, Wingless-type MMTV integration site family member 3 A (WNT3A), p38 inhibitor, glycogen synthase kinase 3β (GSK3β) inhibitor, epidermal growth factor (EGF), nicotinamide, activin-like kinase 5 (ALK5) inhibitor, and Rho-associated protein kinase (ROCK) inhibitor [24, 25]. In this study, we established porcine intestinal organoids using a limited set of growth factors and small molecules, including WNT3A, R-spondin 3, noggin, EGF, ALK5 inhibitor, and ROCK inhibitor. Previous studies have shown that GSK3β suppresses apoptosis and neuronal differentiation in cerebral organoids [26] and increases intestinal organoid size in chicken intestinal organoids [27]. Nicotinamide and p38 inhibitors are known to increase intestinal epithelial cell proliferation while inhibiting differentiation into goblet and enteroendocrine cell types [28]. In this study, CHGA expression was significantly decreased in duodenum-derived organoids compared with that in the native duodenum, and the expression levels of SI and CHGA were decreased in ileum-derived organoids. In contrast, jejunum-derived organoids did not show significant differences compared with the native jejunum. Nevertheless, the developed organoids contained diverse epithelial cell types, including proliferating cells, enteroendocrine cells, goblet cells, and Paneth cells. Although organoids recapitulate the structural and genetic characteristics of the intestine, they could not completely reproduce all genetic features of the small intestinal epithelial cells, which may be due to differences in environmental conditions, such as the gut microbiome and interactions with other cell types, in the absence of microinjection and co-culture systems. The developed organoids could be cultured long term, and various epithelial cell types were detected, such as proliferating cells, goblet cells, enteroendocrine cells, and Paneth cells (Figs. 1b–i and 7c). Our findings suggest that this toxicity model enables clearer interpretation of the roles of p38, GSK, and nicotinamide in cellular signaling and cytotoxic responses, supporting its usefulness for toxicity investigation.

In this study, we examined an organoid model capable of recapitulating the heterogeneity of small intestinal epithelial cells, as evidenced by the presence of diverse epithelial cell types, distinct differentiation patterns, and differentially expressed genes (DEGs) among cell types. scRNA-seq offers novel insights into the mechanisms underlying cell behavior and lineage differentiation, thereby enhancing our understanding of organ development [29]. Previous applications of scRNA-seq in the pig small intestine facilitated the identification of breed-specific immune differentiation during different domestication stages and revealed pathophysiological changes associated with weaning in piglets [30, 31]. However, the application of scRNA-seq in small intestinal organoid research remains limited, with most studies focusing on cellular characterization, the suitability of two-dimensional (2D) culture systems for intestinal function, and viral infection models [32, 33]. Our results demonstrate that the developed organoids comprise various epithelial cell types and mimic the temporal patterns of intestinal growth and differentiation, as indicated by gene expression changes at different time points. Additionally, temporal gene expression patterns were confirmed (Figs. 2 and 3).

Among the hundreds of known mycotoxins, DON and ZEA are the most commonly detected in feed raw materials, and are frequently found together [4]. Previous studies have shown that DON induces immune responses, inhibits protein synthesis, upregulates inflammatory cytokines, disrupts the intestinal barrier, and triggers apoptosis, whereas ZEA causes DNA damage, oxidative stress, and inflammation, leading to impaired growth performance [34–36]. DON-induced disruption of intestinal barrier function has been shown to occur through activation of p38 and c-Jun in epithelial cells [37]. A previous study also showed that DON activates GSK3β in the Wnt signaling pathway and inhibits stemness in porcine enteroids [38]. Our findings demonstrate that DON and ZEA decrease cell viability, alter crypt–villus-like architecture, reduce proliferative cell rates, and disrupt intestinal barrier function, which is consistent with previous reports (Fig. 5). L-WRN organoid medium contains a ROCK inhibitor to suppress caspase-dependent apoptosis and support long-term organoid culture [39, 40]. In the present study, the ROCK inhibitor was included in the L-WRN organoid medium. The apoptosis rates induced by DON and ZEA showed a lower trend compared with the reduction in cell viability, which may be attributed to the protective effects of the ROCK inhibitor in the culture medium. Although ZO-1 is widely recognized as a representative tight junction protein, the loss of other tight junction components, such as occludin (OCLN), has also been reported to be sufficient to increase intestinal permeability. The cellular localization of tight junction proteins is closely associated with intestinal barrier function, and progression to cancer, epithelial–mesenchymal transition (EMT), and inflammation can induce alterations in their cellular localization, ultimately leading to disruption of intestinal barrier function [8, 41, 42]. Our data showed an irregular expression pattern of ZO-1 in the DON- and ZEA-treated groups. However, the molecular mechanisms regulating tight junctions are still not fully understood. Therefore, further studies are needed to elucidate the mechanisms underlying mycotoxin-induced disruption of the intestinal barrier. We assessed nutrient absorption, one of the key functions of the small intestine. Apical-out organoids showed higher absorption efficiency compared to basal organoids. However, this tool could not be applied to direct comparisons of absorption efficiency after DON and ZEA treatment, because increased paracellular permeability allowed nutrient analogs to enter the lumen. This is a limitation of the in vitro model.

The small intestinal epithelium is composed of various secretory cell types, including enteroendocrine, goblet, and Paneth cells. Among these, goblet cells are the most common secretory cells in the small intestine and play a crucial role in protecting against external antigens by forming the mucus layer, which also provides a niche for antimicrobial peptides. Paneth cells secrete Wnt and Notch ligands to maintain the stemness of ISCs and produce antimicrobial peptides [43]. Enteroendocrine cells, including L, X, A, K, I, and D cells, secrete hormones that regulate appetite, gut mucosal growth, nutrient sensing and absorption, glucose metabolism, proliferation, and motility in the small intestine [44]. Despite their critical roles in the small intestine, research on the effects of DON and ZEA on secretory cell types in porcine small intestinal organoids remain limited. In our study, DON and ZEA reduced differentiation into enteroendocrine and goblet cells. However, Paneth cell populations were not altered following DON treatment, whereas ZEA induced an increase in Paneth cell populations. Similarly, previous studies have shown that DON and ZEA are associated with decreased goblet cell numbers in humans and mice [45, 46]. Enteroendocrine and Paneth cells are extremely rare populations in the small intestine; therefore, research on their differentiation in livestock species remains limited. Additionally, interactions among intestinal epithelial cells are highly complex and involve processes such as cellular plasticity and dedifferentiation, which require further investigation. In this context, the ZEA-induced increase in Paneth cell populations observed in our study may reflect, at least in part, an adaptive or protective response to acute toxicity.

## Conclusion

This study developed porcine intestinal organoids and characterized all major types of intestinal epithelial cells. Additionally, the expression patterns of genes related to growth and differentiation were confirmed at different time points. Based on these data, the developed organoid medium was confirmed to be suitable for porcine intestinal organoid culture without the need for additional small molecules. Furthermore, the organoids recapitulated essential intestinal functions, including internal barrier formation and nutrient uptake. Using this model, we examined the cytotoxic effects of DON and ZEA on organoid growth, proliferation, intestinal barrier function, and differentiation. This model can be applied to assess the cytotoxicity of harmful factors to improve pig productivity and represents an attractive in vitro model for studying disease, differentiation, and development. Moreover, it provides an alternative to animal experiments, supporting animal welfare.

## Supplementary Information

Additional file 1: Fig. S1. DEGs and GO analysis according to differentiation.

Additional file 2: Fig. S2. Porcine epidemic diarrhea virus receptor expression in porcine intestinal organoid.

Additional file 3: Table S1. List of primer.

## Data Availability

The data for this study is available from the corresponding author upon reasonable request.

## Abbreviations

CHGA: Chromogranin A
DCLK1: Doublecortin-like kinase 1
DON: Deoxynivalenol
ISCs: Intestinal stem cells
LGR5: Leucine-rich repeat containing G protein-coupled receptor 5
LYZ: Lysozyme
MUC2: Mucin 2
scRNA-seq: Single cell RNA sequencing
SI: Sucrase-isomaltase
ZEA: Zearalenone
ZO-1: Zonula occludens-1
