# Supplementary material for: Dynamic cellular heterogeneity revealed through a time-resolved single-cell atlas: assessment of porcine intestinal organoids as an in vitro model for deoxynivalenol and zearalenone
Source: J Anim Sci Biotechnol. 2026 Jun 3;17:108. doi: 10.1186/s40104-026-01424-9 (PMC13231639; doi:10.1186/s40104-026-01424-9)
Supplement: Supplementary file 1 — Additional file 1: Fig. S1. DEGs and GO analysis according to differentiation. [file 40104_2026_1424_MOESM1_ESM.pdf]

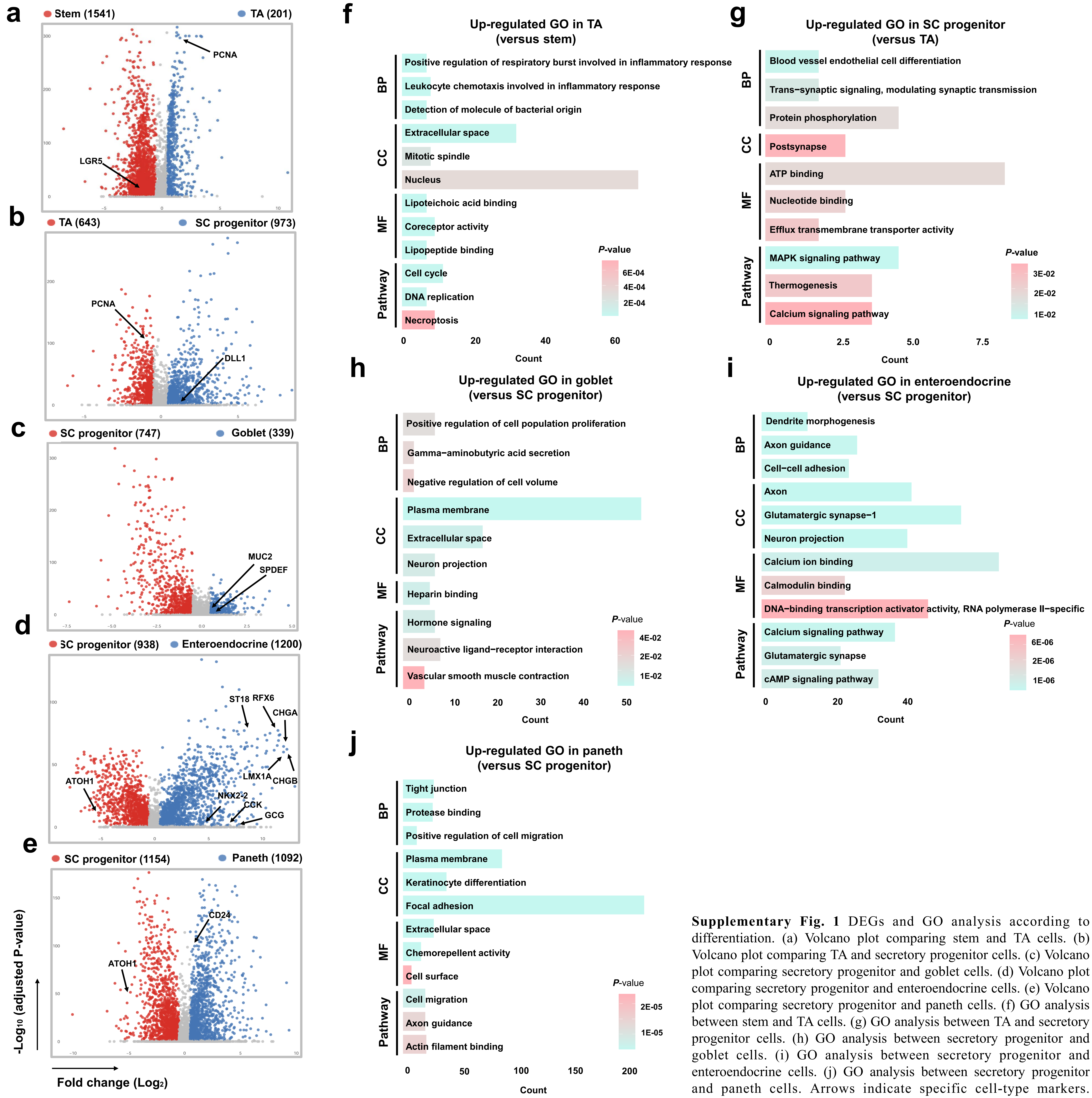

Supplementary Fig. 1 DEGs and GO analysis according to differentiation. (a) Volcano plot comparing stem and TA cells. (b) Volcano plot comparing TA and secretory progenitor cells. (c) Volcano plot comparing secretory progenitor and goblet cells. (d) Volcano plot comparing secretory progenitor and enteroendocrine cells. (e) Volcano plot comparing secretory progenitor and paneth cells. (f) GO analysis between stem and TA cells. (g) GO analysis between TA and secretory progenitor cells. (h) GO analysis between secretory progenitor and goblet cells. (i) GO analysis between secretory progenitor and enteroendocrine cells. (j) GO analysis between secretory progenitor and paneth cells. Arrows indicate specific cell-type markers.
