# Supplementary material for: Dynamic cellular heterogeneity revealed through a time-resolved single-cell atlas: assessment of porcine intestinal organoids as an in vitro model for deoxynivalenol and zearalenone
Source: J Anim Sci Biotechnol. 2026 Jun 3;17:108. doi: 10.1186/s40104-026-01424-9 (PMC13231639; doi:10.1186/s40104-026-01424-9)
Supplement: Supplementary file 2 — Additional file 2: Fig. S2. Porcine epidemic diarrhea virus receptor expression in porcine intestinal organoid. [file 40104_2026_1424_MOESM2_ESM.pdf]

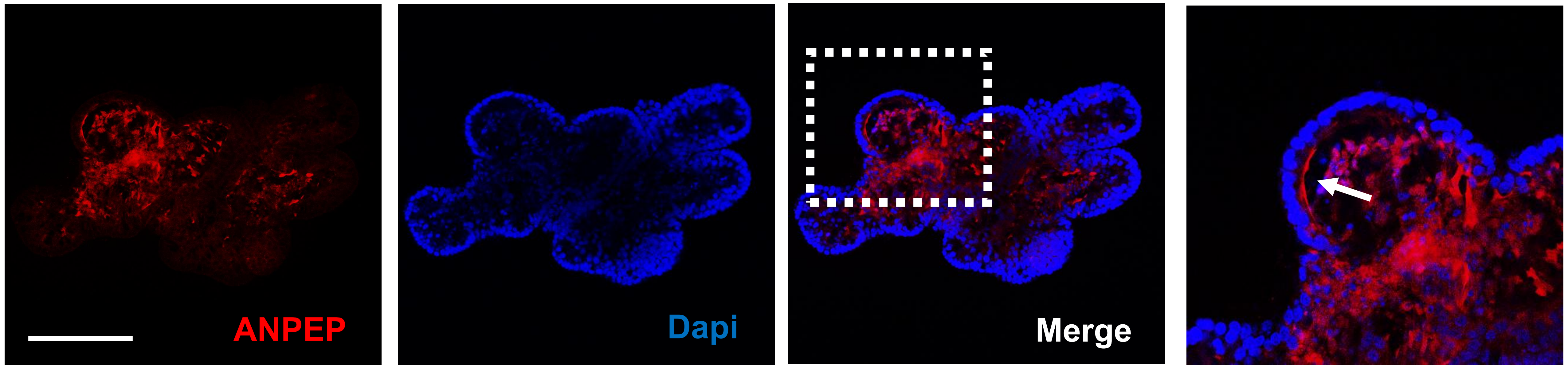

Supplementary Fig. S2 Porcine epidemic diarrhea virus receptor expression in porcine intestinal organoid. Immunostaining of aminopeptidase N (ANPEP) in porcine intestinal organoid. Nuclei were stained with 4',6-diamidino-2-phenylindole (DAPI; blue). Scale bar = 50 μm. The arrow indicate ANPEP protein.
